# Prevalence of depression and suicide ideation in Hong Kong doctors: a cross-sectional study

**DOI:** 10.1038/s41598-021-98668-4

**Published:** 2021-09-29

**Authors:** Amy Pui Pui Ng, Weng Yee Chin, Eric Yuk Fai Wan, Julie Chen, Chak Sing Lau

**Affiliations:** 1 Department of Family Medicine and Primary Care, The University of Hong Kong-Shenzhen Hospital, 1 Haiyuan 1st Rd, Futian District, Shenzhen, 518053 Guangdong Province China; 2 Department of Family Medicine and Primary Care, Li Ka Shing Faculty of Medicine, The University of Hong Kong, 3/F., 161 Main Street, Ap Lei Chau Clinic, Ap Lei Chau, Hong Kong, Hong Kong SAR; 3 Li Ka Shing Faculty of Medicine, Bau Institute of Medical and Health Sciences Education, The University of Hong Kong, 21 Sassoon Road, Pok Fu Lam, Hong Kong, Hong Kong SAR; 4 Department of Pharmacology and Pharmacy, LKS Faculty of Medicine, HKU, 2/F, Laboratory Block, 21 Sassoon Road, Pok Fu Lam, Hong Kong, Hong Kong SAR; 5 Department of Medicine Hong Kong, Li Ka Shing Faculty of Medicine, The University of Hong Kong, Queen Mary Hospital, Room 405B, 4/F, Professorial Block, 102 Pok Fu Lam Road, Pok Fu Lam, Hong Kong, Hong Kong SAR

**Keywords:** Health occupations, Risk factors, Health care, Health policy

## Abstract

Depression amongst physicians can lead to poor individual and institutional outcomes. This study examined the prevalence and factors associated with depression and suicidal ideation amongst doctors in Hong Kong. Doctors who graduated from medical school at the University of Hong Kong between 1995 and 2014 were invited to participate in a survey measuring depressive symptoms, suicidal ideation and thoughts of self-harm, lifestyle behaviours, career satisfaction and socio-demographic characteristics. Data collection occurred between January and April 2016. The prevalence of screened-positive depression was 16.0% and 15.3% of respondents reported having suicidal ideation. Amongst those with positive depression screening scores, less than half reported having a diagnosed mood disorder. Sleeping fewer hours was associated with higher depression scores (*P* < 0.001) and an increased odds of meeting the cut-off for depression (*P* < 0.001). Factors associated with suicidal ideation included being unmarried (*P* = 0.012) and sleeping fewer hours (*P* = 0.022). Hong Kong doctors appear to have high rates of undiagnosed depression, and high levels of depressive symptoms and suicidal ideations. There is a need for greater awareness of the morbidity due to depression and to promote better mental health help-seeking among physicians. Barriers to mental health help-seeking need to be addressed and appropriate resources allocated to reduce suffering.

## Introduction

Depression is a common mental disorder. In 2015, the World Health Organization (WHO) ranked it as the single largest contributor to global disability^1^. Studies have shown that people with depression are twice as likely to die prematurely due to any cause^2^ and have an increased risk for suicide^3^. WHO estimated the global prevalence of depression between 2.6 and 5.9% in 2015^4^. In the workforce, depressed workers are at higher risks of absenteeism, performance deficits and unemployment^5,6^.

A systematic review of 46 physician population studies found that doctors experience higher rates of depression and suicide than the general population^7^. A meta-analysis of 54 studies estimated the prevalence of depression or depressive symptoms amongst resident physicians to be 28.8%^8^, much higher than the WHO global estimate. Like other people with depression, depression in physicians can have a huge impact on their well-being and work performance. Depression has been associated with increased medical errors, decreased ability to handle work-related stress, increased absenteeism, discontinuation of medical training, disruption in personal lives, suicide, and poor quality of life^7,9^. A systematic review of resident physicians found that poor physical health, an unhappy childhood, and stress at work were related to depression. However, factors such as work hours, sleep deprivation, gender, social supports and marital status were not consistent with some studies indicating relationships and others not^7^. Studies have found that specialty choice may affect the risk of depression^10^ and there are strong associations between depression and alcoholism^11,12^, smoking^13,14^, and profession dissatisfaction^15^.

Studies suggest that Chinese physicians have higher rates of depression than the general population. The general population prevalence of depressive symptoms in China has been estimated to range from 31 to 47%^16^, whereas the prevalence in physicians is reported to range between 28.13 and 65.3%^14,17,18^. A study of 2641 physicians in China found that poor self-reported physical health, lengthy working hours, frequent night shifts and lack of regular physical exercise were related to more anxiety and depressive symptoms^17^. In Hong Kong (HK) the population prevalence for depression has been reported to range from 1.5 to 10.7%^19,20^. Although there have been studies examining burnout^21,22^ and stress^23^ in HK doctors, estimates for depression in HK doctors has only been studied among first-year interns where 35.8% of respondents demonstrated abnormal levels of depression as measured by the Depression, Anxiety and Stress Scale^23^.

Thoughts of suicide or harming oneself can occur when a person has depression and may be related to more severe forms of depression^24^. In a 2019 meta-analysis of 25 studies, the standardized mortality rate for suicides among physicians was calculated to be 1.44 (95% CI 1.1.6–1.72, I^2^ = 93.9%) with an overall prevalence of 1.0% for suicide attempts and 17% for suicidal ideation^25^. In 2012, a study of HK physicians reported that of the 226 physicians sampled, none had attempted suicide but 4.9% reported having suicidal ideations^21^, which is higher than the prevalence of 3.5% as observed in a general HK population study^20^. In China, an analysis of 18 physicians who committed suicide from 2004 to 2017 found that work stress and patient-doctor disputes were the most common reasons doctors committed suicide^26^.

Health and psychosocial factors have been shown to be related to retirement intentions of physicians^27^ and to date, these issues have not been explored in existing HK physician manpower surveys^28^.

With a skilled and healthy workforce being the foundation of a robust health care system, local data on the mental health of doctors would provide insight into a poorly investigated area that has implications on both patient care and physician well-being.

### Aims

The aims of this study were to examine the epidemiology of depression and suicidal ideation among HK doctors.

Specific objectives

- To examine the prevalence of depression and suicidal ideations, and to assess depressive symptom severity among doctors in HK.
- To explore the factors associated with depression and suicidal ideation.

Hypotheses

- The prevalence of depression and suicidal ideation in HK doctors will be higher than that in the general HK population and comparable to that of doctors overseas.
- Depression and suicidal ideation will be related to such factors as age, gender, work setting, lifestyle behaviours and job satisfaction.

## Methods

Medical school graduates from the University of Hong Kong between 1995 and 2014 who had a valid email or mailing address (N = 1607) were invited to participate in this cross-sectional study. Data collection occurred between January 29, 2016 to April 15, 2016. Subjects with valid email addressed were contacted by email to complete an online survey in English via SoGo Survey which also tracked the responses electronically. Three reminder emails were sent 14 days apart following the initial invitation. To increase sample size, paper questionnaires were subsequently mailed to graduates with available mailing addresses. Respondents were offered a coffee coupon as an incentive. The survey was voluntary, and response to the survey was taken as implied consent. The institutional review board allowed implied consent as the risk of harm from the survey study was minimal, the population was deemed not vulnerable, and the data collection was fully de-identified and anonymous. Collecting signatures for consent could increase a perceived risk for subject identification and deter potential respondents from completing the survey.

**Figure 1:**
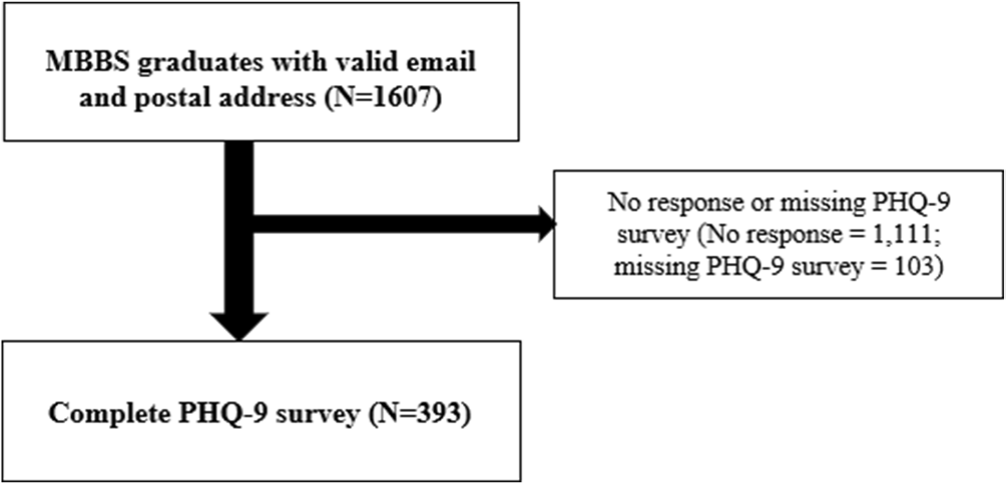
Flow chart showing the sampling and response rates. MBBS, Bachelor of Medicine and Bachelor of Surgery; PHQ-9, Patient Health Questionnaire-9.

In total, 393 (384 by online and 112 by paper survey) respondents completed the PHQ-9 and other instruments shown below. The subject recruitment flow chart is shown in Fig. 1. Institutional Review Board of the University of Hong Kong/ Hospital Authority Hong Kong West Cluster (UW 15-405) approved the study and waived the need for signed informed consent. All research procedures were performed in accordance to the relevant regulations.

### Study instruments

*Patient Health Questionnaire-9* (PHQ-9) was used to calculate the prevalence of depression and suicide intentions. The instrument consists of nine items based on the DSM-V definition for depression. Examples of the items are “having little interest or pleasure in doing things”, “feeling tired or having little energy” and “feeling down depressed and hopeless”. Each item is scored from zero (not at all) to three (nearly every day) with a minimum scores of zero (no depression) to 27 (severe depression)^29^. In this study, a PHQ > 9 was used as the cut-off indicating the presence of depression. Using a cut-off score of > 9, the PHQ-9 has a sensitivity of 80% and specificity of 92% for the diagnosis of depression in HK^30^. PHQ-9 is a reliable and validated survey for depression and has a Cronbach $\alpha$ of 0.89^31^. The presence of suicidal ideation was assessed using the final item of the PHQ-9, “in the past 2 weeks, how often have you been thinking that you would be better off dead or that you want to hurt yourself in some way?” Any positive response indicated the presence of suicidal ideation. A study comparing the endorsement of this single question to endorsement of suicidality in a structured clinical interview found the PHQ-9 suicide item had a specificity of 84% and sensitivity of 69% for identifying suicidality^32^.

*The alcohol use disorders identification test version C* (AUDIT-C) is a screening instrument using three questions to assess drinking behaviour including the frequency of drinking alcohol, the frequency of drinking more than 5 alcoholic units in one occasion, and the average number of alcoholic units consumed on typical day they drink^33^. In accordance to the HK guidelines, scores > 3 indicate a positive screen for at-risk drinking^34^. The scores in this survey were derived from the original questionnaire and adapted to the scoring system of AUDIT-C. Cronbach $\alpha$ for AUDIT-C was previously reported in a range of 0.80–0.91^35,36^.

Items on job satisfaction and lifestyle behaviours were modified from existing doctor questionnaires^37^ and population health surveys from HK^20^. The original survey also included items on burnout and these findings have been published separately^22^.

## Data analysis

Descriptive statistics were used to summarize the socio-demographic characteristics, lifestyle behaviour and career satisfaction of the respondents. Prevalence of depression was calculated by determining the proportion of respondents with PHQ-9 scores > 9. Those who reported several days, more than half the days and nearly every day were included for estimation of the prevalence of suicidal ideation and self-harm for the last question of the PHQ-9. Univariate linear regression was used to evaluate the effect of socio-demographic factors, lifestyle behaviours and career satisfaction on PHQ-9 total scores, PHQ-9 > 9, and PHQ-suicide. If the factors were significantly associated with the outcomes, these factors were considered in the multivariable linear regression models using a forward stepwise selection. Residual normality assumption was examined using Q./Q-plot. Multicollinearity was evaluated using a variance inflation factor. The variance inflation factor showed 1.23, which was below 10, indicating no multicollinearity between the potential factors.

Complete case analysis, using subjects without any missing data only, was conducted as a sensitivity analysis. Missing data for subjects’ characteristics were handled by multiple imputation using the chained equation method. Each missing value was imputed five times based on all characteristics of the participants including age, gender, marital status, having children, current specialty, setting of practice, satisfaction with present job position, satisfaction with being a medical doctor, average sleep per night, hours of work per week, regular exercise, at-risk drinker, current smoker, and PHQ-9 score. Five imputed datasets were generated, and the results were pooled according to the Rubin’s rule^38^.

All significance tests were two tailed and findings with *P*-value < 0.05 were considered statistically significant. All statistical analyses were performed using Stata Version 15.0 (StataCorp LP, College Station, TX, USA).

## Results

### Socio-demographics, lifestyle behaviours and professional satisfaction characteristics

A total of 393 subjects were included for analysis. Data completion rates were over 95.7% for all items aside from drinking habits (89.8%) (see Supplementary Table 1). Missing data in marital status, satisfaction with present job position, average sleep per night, hours of work per week, at-risk drinker were imputed using multiple imputation. Subject characteristics including socio-demographics, lifestyle behaviours and professional satisfaction are shown in Table 1. Mean age of the respondents was 32.8 (SD = 5.4) years and 45% were female. Most respondents indicated they were satisfied with their current job position (78.6%) and with their career choice as a doctor (93.9%), 6.4% reported a previous mood disorder diagnosis, 75.8% performed regular exercise, and 25.8% were classified as at-risk drinkers. Only 2 participants identified as being a current smoker.

**Table 1:** Descriptive statistics on sociodemographic, professional satisfaction and health status.

	Doctors (N = 393)		Doctors (N = 393)
**Socio-demographic**		**Professional satisfaction**	
Age	32.8 ± 5.4	Satisfied with present job position	309 (78.6%)
Gender (female)	177 (45.0%)	Satisfied with being a medical doctor	369 (93.9%)
**Marital status**		**Health status**	
Single, separated and divorced	206 (52.3%)	Hours of work per week	55.1 ± 15.3
Married	187 (47.7%)	Average sleep per night	6.7 ± 1.0
Having children	135 (34.4%)	Regular exercise	298 (75.8%)
**Current specialty**		At-risk drinker	101 (25.8%)
Anaesthesiology/emergency medicine/intensive care	47 (12.0%)	Current smoker	2 (0.5%)
Clinical oncology/dermatology and venerology/internal medicine	73 (18.6%)	PHQ-9 > 9	63 (16.0%)
Pathology/radiology	36 (9.2%)	PHQ-9 Suicide	60 (15.3%)
Family medicine/general practice/community medicine	72 (18.3%)	PHQ-9 Total Score	5.3 ± 5.7
Obstetrics and gynaecology	26 (6.6%)		
Orthopaedic surgery/otorhinolaryngology/surgery/ophthalmology	84 (21.4%)		
Paediatrics	27 (6.9%)		
Psychiatry	28 (7.1%)		
**Setting of practice**			
Public	332 (84.5%)		
Private	61 (15.5%)		
Primary care	67 (17.0%)		

Current smoker (Current smoker vs non-smoker/ex-smoker).

At-risk drinkers were defined if the doctors had 3 or more in AUDIT-C score.

Private Practice (Private Solo/ Private Hospital/Non-government organisation).

Public Practice (University/Government/Hospital Authority/Not applicable).

Regular exercise (5 or more days per week for at least 10 min per day / Any vigorous and moderate physical activities)

All data are represented in mean ± SD or total (%), as appropriate.

### Prevalence of depression and suicidal and self-harm ideations

The prevalence of depression (PHQ-9 > 9) in the overall sample was 16.0% (males 15.8% and females 16.4%). The prevalence of suicide ideations and self-harm thoughts over the past 2 weeks was 15.3%.

### Severity of depressive symptoms

The mean PHQ-9 score was 5.26 (SD = 5.65). 58.78% (n = 231) had PHQ-9 scores of 0–4 (minimal depressive symptoms), 25.19% (n = 99) had scores of 5–9 (mild depressive symptoms), 7.12% (n = 28) had scores of 10–14 (moderately depressive symptoms), 6.11% (n = 24) had scores of 15–19 (moderately severe depressive symptoms) and 2.8% (n = 11) had scores 20–27 (severe depressive symptoms).

### Factors associated with depression, suicidal and self-harm ideations

**Figure 2:**
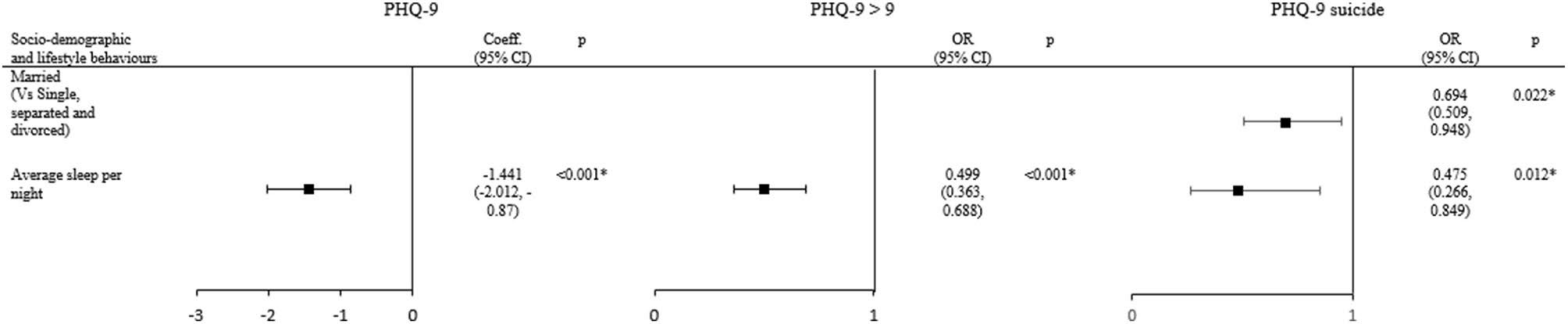
Sociodemographic, professional satisfaction and lifestyle behaviours associated with PHQ-9 score by regression with forward stepwise selection. PHQ-9, Patient Health Questionnaire-9, CI = Confidence Interval; Coeff = Coefficient; OR = Odds Ratio, * Significant with *p*-value < 0.05.

Tables 2, 3, 4 and Fig. 2 shows the results of the regression analyses identifying the factors associated with PHQ-9 total scores, PHQ-9 > 9 and PHQ-9 suicide and self-harm risk using univariate then forward stepwise selection to identify significant associations. Only sleeping fewer hours per night (Coeff =  − 1.441, 95% CI − 2.012 to − 0.870) was associated with higher PHQ-9 scores (Table 2). Using a PHQ-9 cut-off > 9 as a positive screen for depression, only average hours of sleep had a significant odds ratio (OR = 0.499, 95% CI 0.363–0.688) indicating that sleeping 1 h less was associated with a 50% increased odds of having depression (Table 3). PHQ-9 scores and risk of screening positive for depression were not significantly associated with any socio-demographic factors, professional satisfaction, and other lifestyle behaviours such as hours of work, smoking status, regular exercise, and at-risk drinking. Being married reduced the odds of having suicidal ideations and self-harm thoughts by 52.5% (OR = 0.475, 95% CI 0.266–0.849, *P* = 0.012) and sleeping 1 h less increased that odd by 31% (OR = 0.694, 0.509–0.948, *P* = 0.022) (Table 4). Apart from marital status and hours of sleep, no other socio-demographic factors, professional satisfaction or other lifestyle behaviours were significantly associated with suicidal ideations and self-harm thoughts. A sensitivity analysis using the complete case analysis demonstrated similar results compared to the main analysis (Supplementary Tables 2–4).

**Table 2:** Sociodemographic, professional satisfaction and lifestyle behaviour associated with PHQ-9 total score by regression analysis.

Factor†	PHQ-9 score (N = 393)					
	Univariate			Forward stepwise selection		
	Coeff	95% CI	*P*-value	Coeff	95% CI	*P*-value
**Socio-demographic**						
Age	− 0.071	(− 0.176, 0.033)	0.181			
Female (vs male)	0.685	(− 0.442, 1.812)	0.233			
Married (vs single, separated and divorced)	− 1.129*	(− 2.249, − 0.009)	0.048*		NA	
Having children (vs no children)	− 0.952	(− 2.131, 0.227)	0.113			
Private setting of your practice (vs public)	− 1.048	(− 2.595, 0.500)	0.184			
**Current specialty**						
Anaesthesiology/emergency medicine/intensive care	1.891	(− 0.201, 3.984)	0.076			
Clinical oncology/dermatology and venerology/internal medicine	0.746	(− 1.108, 2.599)	0.429		NA	
Pathology/radiology	− 0.028	(− 2.306, 2.250)	0.981			
Family medicine/general practice/community medicine		Reference group				
Obstetrics and gynaecology	1.214	(− 1.339, 3.767)	0.351			
Orthopaedic surgery/otorhinolaryngology/surgery/ophthalmology	0.706	(− 1.086, 2.498)	0.439			
Paediatrics	1.296	(− 1.222, 3.814)	0.312			
Psychiatry	0.337	(− 2.148, 2.823)	0.790			
**Professional satisfaction**						
Satisfied your present job position (vs not satisfied)	− 0.424	(− 1.793, 0.946)	0.543			
Satisfied with being a medical doctor (vs not satisfied)	− 0.875	(− 3.219, 1.469)	0.464		NA	
**Lifestyle behaviours**						
Average sleep per night	− 1.441*	(− 2.012, − 0.870)	< 0.001*	− 1.441*	(− 2.012, − 0.870)	< 0.001*
Hours of work per week	0.048*	(0.011, 0.085)	0.010*			
Current smoker (vs non-smoker/ex-smoker)	5.767	(− 2.106, 13.640)	0.151			
Regular exercise (vs no regular exercise)	− 1.612*	(− 2.914, -0.310)	0.015*		NA	
At-risk drinker	0.727	(− 0.646, 2.101)	0.297			

CI = Confidence Interval; Coeff = Coefficient; NA = Not Applicable.

Current Smoker (Current smoker vs Non-smoker/ex-smoker).

Regular exercise (5 or more days per week for at least 10 min per day / Any vigorous and moderate physical activities).

Private Practice (Private Solo/ Private Hospital/Non-government organisation).

Public Practice (University/Government/Hospital Authority/Not applicable).

At-risk drinkers were defined if the doctors had 3 or more in AUDIT-C score.

* Significant with *p*-value < 0.05.

^†^ Variable in brackets is the reference category for independent variables.

**Table 3:** Sociodemographic, professional satisfaction and lifestyle behaviour associated with PHQ-9 > 9 by regression analysis.

Factor†	PHQ-9 > 9 (N = 393)					
	Univariate			Forward stepwise selection		
	OR	95% CI	*P*-value	OR	95% CI	*P*-value
**Socio-demographic**						
Age	0.961	(0.912, 1.012)	0.133			
Female (vs male)	1.049	(0.611, 1.801)	0.863			
Married (vs single, separated and divorced)	0.503*	(0.289, 0.874)	0.015*		NA	
Having children (vs no children)	0.664	(0.364, 1.210)	0.181			
Private setting of your practice (vs public)	0.325*	(0.113, 0.930)	0.036*			
**Current specialty**						
Anaesthesiology/emergency medicine/intensive care	2.743*	(1.024, 7.344)	0.045*			
Clinical oncology/dermatology and venerology/internal medicine	1.419	(0.535, 3.764)	0.482		NA	
Pathology/radiology	1.600	(0.510, 5.022)	0.421			
Family medicine/general practice/ community medicine		Reference group				
Obstetrics and gynaecology	1.455	(0.399, 5.307)	0.570			
Orthopaedic surgery/otorhinolaryngology/surgery/ophthalmology	1.205	(0.457, 3.182)	0.706			
Paediatrics	2.800	(0.903, 8.684)	0.075			
Psychiatry	1.333	(0.368, 4.837)	0.662			
**Professional satisfaction**						
Satisfied your present job position (vs not satisfied)	0.942	(0.492, 1.805)	0.858			
Satisfied with being a medical doctor (Vs not satisfied)	0.952	(0.314, 2.885)	0.930		NA	
**Lifestyle behaviours**						
Average sleep per night	0.499*	(0.363, 0.688)	< 0.001*	0.499*	(0.363, 0.688)	< 0.001*
Hours of work per week	1.018*	(1.000, 1.036)	0.044*			
Current Smoker (vs non-smoker/ex-smoker)	5.306	(0.328, 85.969)	0.240			
Regular exercise (vs no regular exercise)	0.632	(0.351, 1.140)	0.128		NA	
At-risk drinker	1.503	(0.779, 2.902)	0.222			

CI = Confidence Interval; OR = Odds Ratio; NA = Not Applicable.

Current Smoker (Current smoker vs Non-smoker/ex-smoker).

Regular exercise (5 or more days per week for at least 10 min per day / Any vigorous and moderate physical activities).

Private Practice (Private Solo/ Private Hospital/Non-government organisation).

Public Practice (University/Government/Hospital Authority/Not applicable).

At-risk drinkers were defined if the doctors had 3 or more in AUDIT-C score.

* Significant with *p*-value < 0.05.

^†^ Variable in brackets is the reference category for independent variables.

**Table 4:** Sociodemographic, professional satisfaction and lifestyle behaviour associated with PHQ-9 suicide score by regression analysis.

Factor†	PHQ-9 suicide (N = 393)					
	Univariate			Forward stepwise selection		
	OR	95% CI	*P*-value	OR	95% CI	*P*-value
**Socio-demographic**						
Age	0.967	(0.918, 1.020)	0.218			
Female (vs male)	0.922	(0.529, 1.604)	0.773		NA	
Married (vs single, separated and divorced)	0.433*	(0.244, 0.768)	0.004*	0.475*	(0.266, 0.849)	0.012*
Having children (vs no children)	0.533	(0.282, 1.010)	0.054		NA	
Private setting of your practice (vs Public)	0.562	(0.230, 1.370)	0.205			
**Current specialty**						
Anaesthesiology/emergency medicine/intensive care	2.139	(0.810, 5.651)	0.125			
Clinical oncology/dermatology and venerology/internal medicine	1.242	(0.481, 3.205)	0.654		NA	
Pathology/radiology	0.636	(0.161, 2.511)	0.519			
Family medicine/general practice/community medicine		Reference group				
Obstetrics and gynaecology	1.667	(0.502, 5.531)	0.404			
Orthopaedic surgery/otorhinolaryngology/surgery/ophthalmology	1.400	(0.567, 3.457)	0.466			
Paediatrics	1.217	(0.342, 4.339)	0.762			
Psychiatry	0.840	(0.210, 3.360)	0.805			
**Professional satisfaction**						
Satisfied your present job position (vs not satisfied)	0.784	(0.413, 1.489)	0.457			
Satisfied with being a medical doctor (vs not satisfied)	0.666	(0.239, 1.857)	0.437		NA	
**Lifestyle behaviours**						
Average sleep per night	0.659*	(0.486, 0.894)	0.007*	0.694*	(0.509, 0.948)	0.022*
Hours of work per week	1.011	(0.994, 1.029)	0.210			
Current smoker (vs non-smoker/ex-smoker)	5.627	(0.347, 91.214)	0.224			
Regular exercise (vs no regular exercise)	1.328	(0.673, 2.620)	0.413		NA	
At-risk drinker	1.238	(0.659, 2.327)	0.507			

CI = Confidence Interval; OR = Odds Ratio; NA = Not Applicable.

Current Smoker (Current smoker vs Non-smoker/ex-smoker).

Regular exercise (5 or more days per week for at least 10 min per day/Any vigorous and moderate physical activities).

Private Practice (Private Solo/ Private Hospital/Non-government organisation).

Public Practice (University/Government/Hospital Authority/Not applicable).

At-risk drinkers were defined if the doctors had 3 or more in AUDIT-C score.

* Significant with *P*-value < 0.05.

^†^ Variable in brackets is the reference category for independent variables.

## Discussion

This study found that a significant proportion of HK physicians are depressed (16%) and have thoughts of suicide or self-harm (15.3%). These rates appear to be lower than global estimates for physician depression and suicide ideation^8,18,25,39^, but higher than in the HK general population^19,20^. This is consistent with research that shows that physicians are at a higher risk for depression and suicide^7^. Our initial hypothesis that the prevalence of HK physician would be higher than that of the primary care population of 10.7%^19^, appears to be correct; however, the prevalence of suicidal ideations in this physician sample were much higher than the HK population estimate of 3.5%^20^. Our findings were also higher than those by Siu et al. which found 4.9% of HK doctors had suicidal ideations^21^. This could be a result of the wording of the PHQ-9 suicidality item as the question assesses both self-harm and suicidal ideation together which may result in more positive responses as suicidal ideation and self-harm are not differentiated. However, a systematic review of 61 studies revealed that globally, 17% of physicians have suicidal ideations, which is just slightly higher than our study findings^25^. Moreover, the severity of depression in our sample showed that a majority of those who screened positive for depression had either mild and moderate depression, and there was a small subset (2.8%) that had severe depression consistent with previous depressive study of HK interns^23^. Such high screening levels for potential suicidality and depressive symptoms in HK physicians warrants further exploration.

### Association of lifestyle behaviours with depression and suicidal ideation

In this study population, the only factor significantly associated with higher depression symptom scores and odds of being screened positive for depression was sleeping less hours per night. The relationship between sleep deprivation and depression in physicians has been studied extensively but the evidence on the cause and effect is conflicting and confusing. One possible mechanism for sleep deprivation leading to depressive disorders is explained by a model that describes that fatigue can lead to depression^40^ and there is evidence to suggest that sleep deprivation leads to fatigue^41^. Another mechanism described is that poor sleep impairs emotional regulation, such that people are unable to monitor and evaluate emotions and to adjust to the situation, and this has been shown to lead to depression^42^. However, conflicting evidence exists. Sleep deficit is often a symptom of depression which may imply depression is the cause rather than the effect of sleep deficits; and depression and sleep deficits share similar risk factors and biological factors^7,43–45^.

This study also showed that having less sleep increased the odds of suicidal ideations and self-harm behaviour such that sleeping 1 h less increased the odds of suicidal thoughts by 31%. Sleep deficits is a recognized risk factor for suicidal ideations, but this could be confounded by co-existing depression which is seen as a risk factor and symptom for suicidal thoughts^46,47^. In addition, research supports that sleep loss may lead to impulsivity, thus raising unplanned suicidal behaviour^46^. Others have tried to explain the relationship between sleep and suicidal ideation at a biological level, with serotonin levels appearing to play a role in both constructs^48^. Moreover, the average sleep physicians had per night in this study was 6.7 h, which is in the lower end of the average hours of sleep that adults need (6–9 h)^49^. This may indicate a subset of physicians who are not getting adequate sleep. Sleep deprivation in physicians is related to poor outcomes including increased fatigue and medical errors^50,51^, and worryingly sleep deprived doctors are unable to recognize self-fatigue^50^. The relationship between sleep, suicide, depression and how each can independently affect patient care means that getting adequate sleep in physicians is important to address^50^.

At-risk drinking, smoking, exercise, and work hours were not associated with depression and suicide in this study. Literature reports alcoholism is strongly associated with suicide and depression, which is inconsistent with the results of this study^11,12^. This is possibly because the survey items in the present study were not able to differentiate “at-risk” drinkers to those who may meet the definition for alcohol dependence or problem drinkers. The latter are more likely to use alcohol as a coping mechanism of depression or drink excess alcohol causing significant interference with the daily life to cause depression^12^. Likewise, there is evidence that non-smokers have less depressive symptoms^17^, but this was not seen in this study likely because there were only 2 (out of 393) respondents who identified being a current smoker and thus making it difficult to conclude any relationship. Our study also showed most doctors performed some regular exercise. Exercise has been seen as a protective factor against depression and suicide risks^17,52,53^, but this was not seen in the present study. This could be because different studies use different criteria to quantify exercise and thereby exerting different effects. Interestingly, it has been shown that exercise as an intervention can lead to fewer depressive symptoms and better sleep patterns, which can thereby reduce suicide risk^53^.

The evidence on the relationship between work hours and depression remains divided. Whilst longer work hours has been associated with depression in physicians in some studies^17,54^, this current and other studies have not supported this relationship^7,44^. Our study did not find that longer work hours were associated with greater suicide ideation as in other studies^55,56^. Work hours is only one part of the work demands for physicians whereas other studies have also examined working on shifts, evening calls, dealing with patients, relatives and colleagues, and making mistakes as potential factors which may augment the understanding of the effect of work demands on depression^17,22,57^. Furthermore, research suggest that suicidal thoughts may be less likely due to work demands and more likely due to personal and family problems, which we will discuss in the next section^58^.

### Sociodemographic factors associations with depression and suicidal ideations

Of the sociodemographic factors, only marriage was found to be a protective factor in this study against suicide ideations and self-harm. Research has shown that marriage is protective for depression: Both men and women have less distress and depression when married^19^ and being single is a well-documented risk factor for suicide, especially in women^59–61^. Marriage often reflects an effective family support system, which can have protective effects against self-harm behaviours and depression^58,60^. However, the results of this study are confusing with marriage only being associated with suicidal ideations and self-harm but not with depression. Results from other studies do not support the relationship between marriage and depression^18,44^ suggesting that marital status alone may not be enough to explain the relationship and perhaps other factors such as marital satisfaction, marital problems or family satisfaction may play a role^62,63^. In the Chinese culture, positive family relationships are considered an important factor for mental well-being. Family harmony is a measure of how well the family functions together including how well they accommodate each other and have successful daily interactions^63^. Research has shown that for families in HK who reported low family harmony had stronger associations for depressive symptoms^63^. Future research may want to explore how family harmony plays a role in physician depression and suicide ideations and self-harm behaviours.

Age was not found to be significantly associated with depression or suicidality in this study. This is consistent with a systematic review of 46 studies which found that age is not associated with depression^7^. Some studies suggest that older age increases suicide risk which may be related to a loss of connection with colleagues following retirement leading to increased distress^64,65^. However, our sample did not include older physicians and the lack of significance could have been because of the relatively narrow age range of our sample, who were all within 20 years of graduation.

There is a large body of evidence indicating women are at greater risk for depression both globally and in HK^19,66^. Studies have also shown that female physicians are at greater risk for depression and suicide^25,54^. In our sample, gender was not a significant factor, similar to observations in other physician studies^7,44^. In HK, higher-income families and in particular in families where the wife has a high-income job, hiring a live-in domestic helper to assist to what traditional Chinese people see as “women’s tasks” is common^67^. These at-home responsibilities are often perceived as a possible reason for increased risk of depression and suicide in women physician^25^. With easier accessibility and affordability of full-time domestic help in HK, many of the ‘at-home’ responsibilities that are typically are borne by women are reduced and may potentially reduce the risk of depression and suicide in female physicians. Conversely, there may be other factors causing the relatively higher rates of depression and suicidal ideation in men, and the reasons for their elevated risks need further investigation.

Choice of specialty was not a significant factor associated with depression and suicidality in our sample. Whilst some studies have found that specialty may have an effect, the evidence remains inconclusive that any one particular specialty may be associated with a greater risk of depression and suicide intention^10,25,44^. In our study, sample sizes were not large enough to perform subgroup analyses by individual specialties.

### Professional satisfaction association with depression and suicidal ideations

Our study did not reveal any significant associations between job satisfaction and depression, despite 485 studies in a meta-analysis indicating that there is a strong relationship between low job satisfaction, burnout, and increased risk of depression^15^. The respondents in this study expressed high levels of job satisfaction as measured by these two items: “how satisfied are you with your current job position (78.6% satisfied or very satisfied) and “how satisfied are you with being a medical doctor” (93.9% satisfied or very satisfied). Our previous study examining burnout within the same cohort also did not observe any association between career satisfaction and burnout; although an earlier HK physician study did^21,22^. In that earlier study, only 51.4% expressed satisfaction with their job, which may reflect a difference in our sample cohort as only public sector doctors were included in their sample and this current study included doctors working in both private and public sector settings.

### Help-seeking behaviour

Less than half of those who screened positive for depression reported having a diagnosis of a mood disorder indicating a large proportion of HK doctors with depression may be unaware of their mental health problem or are not seeking appropriate care from a mental health professional despite recognizing their mental illness. This is consistent with a previous HK study showing that doctors tend to treat themselves rather than seek help^68^. According to research, physicians face psychological barriers to seeking care for their mental health issues, including feelings of shame and embarrassments, the notion that doctors should appear healthy, and the belief that mental illness is a weakness^69^. Studies also have suggested that it is hard for doctors to adopt the “role reversal” and become a patient^69^. This problem is not unique to HK, and it is a global issue that needs to be addressed^70,71^.

### Limitations

There are several limitations in this study. First, with a response rate of just 24.5%, the findings may be susceptible to response bias: physicians who are more depressed may either be more likely to respond because the topic is relevant or less likely to respond because they are more indifferent. Second, despite having two medical schools and foreign-trained doctors in HK, this study only sampled graduates from one medical school and therefore the findings might not be generalizable to the entire HK doctor population. We studied from a single medical school because pragmatically, we had the contact information of these graduates, which accounted for roughly half of all local graduates^72^. Third, not all age groups were represented in this study as the sample were all within 20 years of graduation, and the age of the participants ranged only between 24 and 45 years old. However, because sampling was adequate from the range of ages across the study sample, it still allowed for relevant relationships to be made. Moreover, in HK, doctors in the private sector comprise of about 48.9% of all doctors, but we only sampled 15.5% of private doctors^73^. This can be explained by the likelihood that more recent graduates are undergoing training which is carried out in the public sector in HK. Lastly, we are unable to establish whether the associations are causally associated because the survey was cross-sectional.

## Conclusion

Doctors in HK are at relatively high risk of having depression and suicidal thoughts; however, many doctors do not seek mental health care or get diagnosed. With such high levels of mental morbidity, better systems are needed to support doctors to better self-care and reduce barriers to seeking help. From our findings, encouraging better self-care in particular better sleep and exercise may be helpful for HK doctors. Lack of sleep can impact patient care directly and indirectly through its effect on increased risk of depression and suicidal risk. Healthcare institutions could enable better physician self-care by optimizing work schedules and promoting wellness education. Implementing well-being curricula into medical school to encourage students develop better self-awareness and self-care might help enhance resilience or reduce the stigma of mental health help-seeking later in their careers.

Strategies to reduce the barriers to accessing mental health care could be trialed such as introducing peer support or counselling schemes and encouraging all physicians to have their own family doctor. Some countries have addressed barriers to help-seeking by introducing doctor-specific health initiatives with staff and resources dedicated to identifying and helping doctors with health problems. Doctors could be screened for depression throughout their career to identify those who may require mental health support. Physician organizations could do more to educate physicians who care for other doctors about maintaining the right balance between respecting their patient's position as a medical professional and listening to their views about their treatment plan, while also standing firm on what is best for the patient’s health.

Fortunately, despite high mental morbidity, work satisfaction levels remain relatively high amongst HK doctors and could potentially act as a protective factor preventing doctors from leaving the profession or opting for early retirement.

## Supplementary Information

Supplementary Information.

## Abbreviations

AUDIT – C: Alcohol use disorders identification test version C
HK: Hong Kong
PHQ-9: Patient health questionnaire-9
WHO: World Health Organization
