# Supplementary material for: Prevalence of depression and suicide ideation in Hong Kong doctors: a cross-sectional study
Source: Sci Rep. 2021 Sep 29;11:19366. doi: 10.1038/s41598-021-98668-4 (PMC8481547; doi:10.1038/s41598-021-98668-4)
Supplement: Supplementary file 1 — Supplementary Information. [file 41598_2021_98668_MOESM1_ESM.pdf]

Prevalence of depression and suicide ideation in Hong Kong doctors: a cross-sectional study

Supplementary Information

Authors: Amy Pui Pui Ng, Weng Yee Chin, Eric Yuk Fai Wan, Julie Chen, Chak Sing Lau

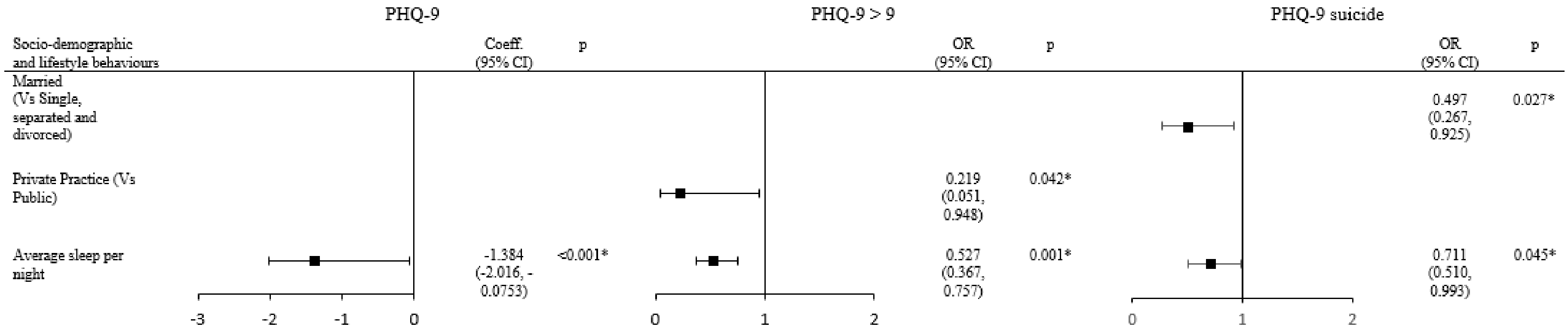

Supplementary Fig. 1 Sociodemographic, professional satisfaction and lifestyle behaviours associated with PHQ-9 score by regression with forward stepwise selection for complete cases.

PHQ-9, Patient Health Questionnaire-9, CI = Confidence Interval; Coeff = Coefficient; OR = Odds Ratio

* Significant with p-value < 0.05

Supplementary Table 1. Data completion rate for sociodemographic status, professional satisfaction, health status

**Total**	Doctors (N =393)
**Socio-demographic**	
Age	100.0% (393)
Gender	100.0% (393)
Marital Status	99.7% (392)
Having Children	100.0% (393)
Current specialty	100.0% (393)
Setting of practice	100.0% (393)
**Professional satisfaction**	
Satisfied with present job position	99.7% (392)
Satisfied with being a medical doctor	100.0% (393)
**Health status**	
Average sleep per night	99.2% (390)
Hours of work per week	98.5% (387)
Regular exercise	100.0% (393)
At-risk drinker	89.8% (353)
Current Smoker	100.0% (393)
Depression	100.0% (393)
PHQ-9 Suicide	100.0% (393)
PHQ-9 Total Score	100.0% (393)

Notes:

PHQ = Patient Health Questionnaire

Supplementary Table 2: Sociodemographic, professional satisfaction and lifestyle behaviours associated with PHQ-9 total score by regression analysis for complete cases.

Factor†	PHQ-9 score (N = 328)					
	Univariate			Forward stepwise selection		
	Coeff.	95% CI	P-value	Coeff.	95% CI	P-value
**Socio-demographic**						
Age	-0.093	(-0.205, 0.020)	0.106			
Female (Vs Male)	0.212	(-1.030, 1.454)	0.738			
Married (Vs Single, separated and divorced)	-1.440*	(-2.668, -0.211)	0.022*		NA	
Having children (Vs No children)	-1.441*	(-2.745, -0.138)	0.030*			
Private setting of your Practice (Vs Public)	-1.562	(-3.234, 0.109)	0.067			
Current specialty						
Anaesthesiology/ Emergency Medicine/ Intensive Care	1.924	(-0.435, 4.283)	0.110			
Clinical Oncology/ Dermatology and Venerology/ Internal Medicine	0.639	(-1.415, 2.693)	0.541		NA	
Pathology/ Radiology	0.456	(-2.126, 3.037)	0.729			
Family Medicine/ General Practice/ Community Medicine		reference group				
Obstetrics & Gynaecology	1.318	(-1.448, 4.083)	0.349			
Orthopaedic Surgery/ Otorhinolaryngology/ Surgery/ Ophthalmology	0.926	(-1.026, 2.878)	0.351			
Paediatrics	1.539	(-1.319, 4.397)	0.290			
Psychiatry	0.839	(-1.926, 3.604)	0.551			
**Professional satisfaction**						
Satisfied your present job position (Vs Not satisfied)	-0.362	(-1.904, 1.181)	0.645		NA	
Satisfied with being a medical doctor (Vs Not satisfied)	-2.000	(-4.863, 0.863)	0.170			
**Lifestyle behaviours**						
Average sleep per night	-1.384*	(-2.016, -0.753)	<0.001*	-1.384*	(-2.016, -0.753)	<0.001*
Hours of work per week	0.045*	(0.006, 0.085)	0.025*			
Current Smoker (VS non-smoker/ex-smoker)	5.687	(-2.233, 13.608)	0.159			
Regular exercise (Vs no regular exercise)	-1.710*	(-3.171, -0.249)	0.022*		NA	
At-risk drinker	1.089	(-0.312, 2.490)	0.127			

Notes:
CI = Confidence Interval; Coeff = Coefficient; NA = Not Applicable
Current Smoker (Current smoker vs Non-smoker/ex-smoker)
Regular exercise (5 or more days per week for at least 10 minutes per day / Any vigorous and moderate physical activities)
Private Practice (Private Solo/ Private Hospital/Non-government organisation)
Public Practice (University/Government/Hospital Authority/Not applicable)
At-risk drinkers were defined if the doctors had 3 or more in AUDIT-C score.
* Significant with p-value < 0.05
† Variable in brackets is the reference category for independent variables

Supplementary Table 3. Sociodemographic, professional satisfaction and lifestyle behaviours associated with PHQ-9 >9 by regression analysis for complete cases.

Factor†	PHQ-9 >9 (N = 328)					
	Univariate			Forward stepwise selection		
	OR	95% CI	P-value	OR	95% CI	P-value
**Socio-demographic**						
Age	0.954	(0.902, 1.010)	0.105		NA	
Female (Vs Male)	0.896	(0.491, 1.637)	0.721			
Married (Vs Single, separated and divorced)	0.436*	(0.234, 0.811)	0.009*			
Having children (Vs No children)	0.502	(0.247, 1.023)	0.058			
Private setting of your Practice (Vs Public)	0.181*	(0.043, 0.768)	0.020*	0.219*	(0.051, 0.948)	0.042*
Current specialty						
Anaesthesiology/ Emergency Medicine/ Intensive Care	2.839	(0.917, 8.789)	0.070		NA	
Clinical Oncology/ Dermatology and Venerology/ Internal Medicine	1.333	(0.433, 4.107)	0.616			
Pathology/ Radiology	1.920	(0.532, 6.933)	0.319			
Family Medicine/ General Practice/ Community Medicine		reference group				
Obstetrics & Gynaecology	1.325	(0.302, 5.811)	0.709			
Orthopaedic Surgery/ Otorhinolaryngology/ Surgery/ Ophthalmology	1.338	(0.457, 3.920)	0.595			
Paediatrics	3.533	(0.994, 12.563)	0.051			
Psychiatry	1.860	(0.473, 7.314)	0.375			
**Professional satisfaction**						
Satisfied your present job position (Vs Not satisfied)	0.901	(0.434, 1.869)	0.779		NA	
Satisfied with being a medical doctor (Vs Not satisfied)	0.788	(0.216, 2.869)	0.718			
**Lifestyle behaviours**						
Average sleep per night	0.507*	(0.356, 0.722)	<0.001*	0.527*	(0.367, 0.757)	0.001*
Hours of work per week	1.015	(0.996, 1.035)	0.114			
Current Smoker (VS non-smoker/ex-smoker)	5.520	(0.340, 89.703)	0.230		NA	
Regular exercise (Vs no regular exercise)	0.592	(0.307, 1.143)	0.118			
At-risk drinker	1.861	(0.990, 3.497)	0.054			

Notes:
CI = Confidence Interval; Coeff = Coefficient; OR = Odds Ratio; NA = Not Applicable
Current Smoker (Current smoker vs Non-smoker/ex-smoker)
Regular exercise (5 or more days per week for at least 10 minutes per day / Any vigorous and moderate physical activities)
Private Practice (Private Solo/ Private Hospital/Non-government organisation)
Public Practice (University/Government/Hospital Authority/Not applicable)
At-risk drinkers were defined if the doctors had 3 or more in AUDIT-C score.
* Significant with p-value < 0.05
† Variable in brackets is the reference category for independent variables

Supplementary Table 4. Sociodemographic, professional satisfaction and lifestyle behaviours associated with PHQ-9 suicide score by regression analysis for complete cases.

Factor†	PHQ-9 suicide (N = 328)					
	Univariate			Forward stepwise selection		
	OR	95% CI	P-value	OR	95% CI	P-value
**Socio-demographic**						
Age	0.976	(0.924 , 1.030)	0.374		NA	
Female (Vs Male)	0.754	(0.414 , 1.372)	0.355			
Married (Vs Single, separated and divorced)	0.446*	(0.242 , 0.820)	0.009*	0.497*	(0.267, 0.925)	0.027*
Having children (Vs No children)	0.537	(0.270 , 1.070)	0.077		NA	
Private setting of your Practice (Vs Public)	0.493	(0.186 , 1.302)	0.153			
Current specialty						
Anaesthesiology/ Emergency Medicine/ Intensive Care	2.388	(0.803 , 7.097)	0.117			
Clinical Oncology/ Dermatology and Venerology/ Internal Medicine	1.286	(0.445 , 3.711)	0.642		NA	
Pathology/ Radiology	0.891	(0.212 , 3.741)	0.875			
Family Medicine/ General Practice/ Community Medicine		reference group				
Obstetrics & Gynaecology	2.063	(0.581 , 7.323)	0.262			
Orthopaedic Surgery/ Otorhinolaryngology/ Surgery/ Ophthalmology	1.677	(0.630 , 4.466)	0.301			
Paediatrics	1.748	(0.455 , 6.709)	0.416			
Psychiatry	0.707	(0.136 , 3.688)	0.681			
**Professional satisfaction**						
Satisfied your present job position (Vs Not satisfied)	0.735	(0.367 , 1.470)	0.384		NA	
Satisfied with being a medical doctor (Vs Not satisfied)	0.400	(0.133 , 1.203)	0.103			
**Lifestyle behaviours**						
Average sleep per night	0.668*	(0.483 , 0.926)	0.015*	0.711*	(0.510, 0.993)	0.045*
Hours of work per week	1.015	(0.996 , 1.034)	0.114			
Current Smoker (VS non-smoker/ex-smoker)	5.269	(0.324 , 85.584)	0.243		NA	
Regular exercise (Vs no regular exercise)	1.331	(0.634 , 2.796)	0.450			
At-risk drinker	1.410	(0.745 , 2.668)	0.291			

Notes:
CI = Confidence Interval; Coeff = Coefficient; OR = Odds Ratio; NA = Not Applicable
Current Smoker (Current smoker vs Non-smoker/ex-smoker)
Regular exercise (5 or more days per week for at least 10 minutes per day / Any vigorous and moderate physical activities)
Private Practice (Private Solo/ Private Hospital/Non-government organisation)
Public Practice (University/Government/Hospital Authority/Not applicable)
At-risk drinkers were defined if the doctors had 3 or more in AUDIT-C score.
* Significant with p-value < 0.05
† Variable in brackets is the reference category for independent variables
